# Percutaneous Ablation of Hepatic Tumors at the Hepatocaval Confluence Using Irreversible Electroporation: A Preliminary Study

**DOI:** 10.3390/curroncol29060316

**Published:** 2022-05-31

**Authors:** Tiankuan Li, Wei Huang, Zhiyuan Wu, Yong Wang, Qingbing Wang, Ziyin Wang, Qin Liu, Jingjing Liu, Shenjie Wang, Xiaoyi Ding, Zhongmin Wang

**Affiliations:** 1 Department of Interventional Radiology, Ruijin Hospital, Shanghai Jiao Tong University School of Medicine, Shanghai 200025, China; ltk12359@rjh.com.cn (T.L.); hw11475@rjh.com.cn (W.H.); wuzhiyuan@shsmu.edu.cn (Z.W.); wqb12049@rjh.com.cn (Q.W.); wzy11837@rjh.com.cn (Z.W.); lq11836@rjh.com.cn (Q.L.); ljj11818@rjh.com.cn (J.L.); wsj12568@rjh.com.cn (S.W.); 2 Department of Interventional Radiology and Vascular Surgery, Zhongda Hospital, Southeast University, Nanjing 210009, China; 101012415@seu.edu.cn

**Keywords:** irreversible electroporation, hepatocaval confluence, perivascular, tumor ablation

## Abstract

Background: Tumors at the hepatocaval confluence are difficult to treat, either surgically or ablatively. Methods: A retrospective longitudinal study on patients ineligible for thermal ablation who underwent computed tomography-guided IRE for hepatic tumors at the hepatocaval confluence was conducted. Factors analyzed included patient and tumor characteristics, IRE procedure details, treatment-related complications, and prognosis. Results: Between 2017 and 2021, 21 patients at our institute received percutaneous IRE. Of the 38 lesions, 21 were at the hepatocaval confluence. Complete ablation was achieved in all cases. Local and distant recurrence was observed in 4.8% (1/21) and 42.6% (9/21) of the ablated tumors, respectively. All postcava remained perfused at follow-up, except for 1 (4.8%) hepatic vein near the lesion found to be temporarily occluded and restored within 1 month. The ratio of the maximum diameter of ablation area at 1, 3, and 6 months post procedure compared to that immediately after IRE was 0.68 (0.50–0.84), 0.49 (0.27–0.61), and 0.38 (0.25–0.59), respectively. Progression-free survival of the patients with recurrence was 121 (range, 25–566) days. Four (19.0%) patients died at the end of follow-up with median overall survival of 451.5 (range, 25–716) days. Conclusions: IRE could be a safe and effective treatment for hepatic tumors at the hepatocaval confluence. This article provides valuable prognostic data; further clinical research is needed for better prognosis.

## 1. Introduction

Hepatocaval confluence is the structure where three hepatic veins flow into the hepatic segment of the inferior vena cava. Hepatic tumor at the hepatocaval confluence is deep and adjacent to large blood vessels (e.g., inferior vena cava, hepatic vein, etc.). Surgical resection may cause severe trauma, loss of liver function, and massive bleeding [1].

Image-guided percutaneous local ablation of hepatic tumors is an established treatment option when surgical resection is not feasible. When the tumor is near important blood vessels, bile duct, or pancreatic duct, heat-based ablation (e.g., radiofrequency ablation (RFA), microwave ablation (WMA), cryoablation, high intensity-focused ultrasound, or laser interstitial thermotherapy) is considered an alternative. Heat sink effect of the blood vessels may influence the treatment outcome of heat-based ablation effect, making incomplete ablation highly possible. Previous work showed that the presence of blood vessels >3 mm near the tumor decreased tumor necrosis rate to <50% [2] When the diameter of the vessel was increased to 5 mm, viable perivascular tissue indicative of heat sink effect was identified in 100% of the studied veins [3]. Therefore, the existence of the hepatic vein and inferior vena cava near the tumor will inevitably affect the success rate of tumor ablation at the hepatocaval confluence. Damage to hollow organs, such as blood vessels, bile ducts, pancreatic ducts, and gallbladder, may lead to complications [4,5,6] and further causes incomplete ablation [7]. There are two main difficulties in the heat-based ablation of tumor at the hepatocaval confluence. First, the heat sink effect of adjacent large vessels is prominent, and vessels >5 mm are prone to incomplete tumor ablation or local tumor progression [3]. Second, due to deep tumor location and complex anatomical structures, the procedure of puncturing and ablating may also cause damage [8].

Irreversible electroporation (IRE) is a novel non-heat-based tumor ablation technique when thermal ablation is unsafe or less effective. Unlike heat-based ablation that induces cell death through coagulation necrosis, IRE creates permanent nanoscale perforations on tumor cells by releasing high-pressure electric pulses, disrupting intracellular homeostasis, and causing programmed cell death [9]. Moreover, IRE rarely generates heat and is hardly affected by external heat or the heat sink effect [10]. The great promise of IRE is that the extracellular matrix is left unperturbed, thus sparing the structural integrity of surrounding structures, such as the hepatic artery, hepatic vein, portal vein, and intrahepatic bile duct [11]. IRE may also damage both tumor blood vessels and cells while preserving normal blood vessels to facilitate complete tumor ablation, which has been demonstrated in preclinical studies [12,13,14]. Although IRE is a viable treatment for hepatic tumors at the hepatocaval confluence, its feasibility, complications, and prognosis have not yet been confirmed in clinical setting.

The purpose of this study is to investigate the safety and efficacy of IRE in treating hepatic tumors at the hepatocaval confluence.

## 2. Materials and Methods

This retrospective study was approved by the Institutional Review Board of Ruijin Hospital, Shanghai Jiao Tong University, School of Medicine on 28 January 2021.

### 2.1. Patients

This retrospective study included consecutive patients with hepatic tumors undergoing IRE procedures between February 2017 and December 2021. All primary tumors were confirmed pathologically, and malignancy of the liver lesions was assessed by multiphase contrast-enhanced computed tomography (CT) and/or magnetic resonance imaging (MRI) within 2 weeks before IRE. Diagnosis and therapeutic decisions were made by a group of interventional radiologists and liver surgeons. Inclusion criterion was lesions up to 3 cm near the hepatocaval confluence. The lesions were defined to be perivascular if they were within 0.5 cm of the major hepatic veins. Patients with cardiac arrhythmias, infection, or uncorrectable coagulopathy were excluded.

### 2.2. IRE Procedure

All procedures were performed using the NanoKnife (AngioDynamics, Latham, NY, USA) system by an experienced team comprising an interventional radiologist, an anesthetist, an itinerant nurse, and a trained machine operator. General anesthesia with neuromuscular blockade was mandatory to minimize unwanted muscular contraction. The pulses were delivered using electrocardiography gating during the refractory phase after myocardial depolarization to minimize the risk of cardiac arrhythmia. A median of 4 (range, 2–4) electrodes were used for each session. IRE electrodes were percutaneously inserted in parallel at 1.5–2.2 cm apart (median, 1.9 cm) under CT guidance in accordance with the manufacturer’s guidance. Median exposure length of electrodes was 2.25 cm (range, 2.0–3.0 cm). Initially, test pulses (10 pulses of 1800 V/cm for 70 μs) were delivered followed by the delivery of 90 target electrical pulses of 1500–3000 V at 20–50 A. Re-intervention was performed if the first treatment was aborted before 90 pulses. Electrode distribution was sited to build an ablation zone encompassing the target lesion and rim of surrounding tissue. Pull-back technique and electrode replacement were performed when necessary. For lesions away from important structures, the expert team discussed between IRE and other ablation methods and asked the patients to approve the final treatment plan. After the procedure, patients were transferred to the interventional ward for close monitoring, and each patient was requested to stay in bed for six hours.

### 2.3. Assessment of Treatment Response and Patient Follow-Up

Immediately after IRE procedure, contrast-enhanced CT of the upper abdomen was performed to assess whether ablation was complete and to detect procedure-related complications (e.g., hemoperitoneum, pneumothorax, pleural effusion, and biliary obstruction). Tumor ablation was considered successful if no arterial hypervascularity or washout in portal venous/delayed phase was seen. All patients underwent contrast-enhanced CT or MRI for lesion evaluation at 1, 3, and 6 months post procedure and every 3 months thereafter. Residual disease or incomplete ablation was defined as the presence of tumor adjacent to the ablation site on CT at 1-month follow-up. Recurrent disease was defined as the appearance of new lesions where the original lesion was absent (<0.5 cm) on contrast-enhanced CT or MRI. The lesions adjacent to the ablation site were termed local recurrence, and those distant from the ablation site were termed distant recurrence. Progression-free survival (PFS) was defined as the time elapsed between the last IRE procedure and tumor progression or death. Overall survival (OS) was from the time of IRE procedure to the time of death from any cause or the end of follow-up.

### 2.4. Outcome Measures and Statistical Analysis

The major procedure-related complications were assessed in accordance with the Cardiovascular and Interventional Radiological Society of Europe (CIRSE) recommendations for evaluation of complications. According to this classification, Grade 1 complications could be solved within the same session during the procedure without additional therapy, post-procedure sequelae, or deviation from the normal post-therapeutic course. Grade 2 complications caused prolonged observation including overnight stay (as a deviation from the normal post-therapeutic course within 48 h) without additional post-procedure therapy or sequelae. All CT and MRI images were assessed by experienced abdominal radiologists in consensus. Data were presented as mean of the measured data. The maximum diameter was used to evaluate the tumor size and ablation zone given tumors are usually oval. Continuous data were expressed as median and range, and categorical variables were expressed as proportion and frequency.

## 3. Results

Of the 38 lesions in 21 patients included in this study, 21 hepatic tumors were detected at the hepatocaval confluence (Table 1). Median age was 58 years (range, 41–83 years). Median body mass index was 22.9 (range, 15.6–26.0). One hepatic tumor was treated per patient (range, 1–5). For underlying diseases, cirrhosis was diagnosed in 9 (42.9%) patients, hypertension in 11 (52.4%), diabetes mellitus in 3 (14.3%), and heart disease in 4 (19.0%). The patients showed acceptable liver function as 20 (95.2%) of them were diagnosed to be Child-Pugh Class A and 1 (4.8%) Class B.

All primary tumors were confirmed pathologically. Although most hepatic tumors could not be confirmed by tissue diagnosis, the imaging characteristics and clinical findings all pointed to malignancy. Tumor types treated with IRE included hepatocellular carcinoma (HCC; *n* = 9; 42.9%), cholangiocarcinoma (*n* = 2; 9.5%), intestinal cancer liver metastases (*n* = 4; 19.1%), gastric carcinoma liver metastases (*n* = 3; 14.3%), and pancreatic carcinoma liver metastases (*n* = 3; 14.3%).

Of the 21 patients, 8 had lesions distant from the hepatocaval confluence, and the median number of the lesions was 1.5 (range, 1–4). The majority of tumors at the hepatocaval confluence were small with the maximum diameter being 1.81 cm (range, 1.31–4.04 cm). Among them, 12 (57.1%) lesions were 1.0–2.0 cm, 5 (23.8%) were 2.01–3.0 cm, and only 4 (19.1%) were >3.0 cm (Table 2). Preoperative images showed that most lesions were located in the right lobe of the liver, with 8 (38.1%) in Segments 8, 7 (33.3%) in Segment 7, 2 (9.5%) in Segment 4a, and 1 (4.8%) in Segment 2. The remaining 3 (14.3%) lesions were located in the junctional region of the segments, with 2 between S4a and S8 and 1 between S7 and S8. In addition, the distance from the tumor margin to the hepatocaval confluence or the closest major hepatic vein was measured on preoperative imaging. The median distance to the hepatocaval confluence was 0.50 cm (range, 0.10–1.72 cm), with 11 (52.4%) lesions ≤0.5 cm, 4 (9.5%) between 0.50 cm and 1.00 cm, and 6 (28.6%) between 1.0 cm and 2.0 cm. All hepatic tumors were close to the major hepatic vein at a median distance of 0.15 cm (range, 0.10–0.45 cm), and this explained why IRE was preferred over thermal ablation. 

The ablation zone was evaluated immediately after IRE using contrast-enhanced CT or MRI. The median maximum diameter of ablation zone was 3.87 cm (range, 2.85–6.55 cm), and 47.6% (10/21) of the ablated zones were 3.01–4.00 cm, 2 (9.5%) at 2.01–3.00 cm, 5 (23.8%) at 4.01–5.00 cm, and 4 (19.0%) > 5.01 cm. IRE ablation zone (tumor size) was 2.83 cm (range, 1.4–3.2 cm).

The number of electrodes, percutaneous insertion route, exposure length, and spacing were determined by the expert team according to the lesion size (Table 3). The voltage was 1500–3000 V, pulse duration was 70 μs, and number of pulse was 90–200. During the procedure, successful energy release of IRE was determined by the real-time impedance change in the ablation range (the current difference between the ablation area before and after energy release was 8–12 A). When complete ablation could not be achieved during a single ablation session, pull-back technique (*n* = 14; 66.7%) or electrode replacement (*n* = 6; 28.6%) was then performed. All procedures were successfully completed without arrhythmia or severe bleeding noticed. Four patients with 6 lesions received IRE for tumors distant from the hepatocaval confluence, and another four received thermal ablation (3 patients having 9 lesions received RFA and 1 having 2 lesions received WMA).

According to the CIRSE recommendations for evaluation of complications (Table 4), 3 (14.3%) patients developed fever, and 2 had different levels of pain (one grade 2 and the other grade 3 as per the numeric rating scale (NRS)). Contrast-enhanced CT performed immediately after the procedure revealed pleural effusion in 11 (52.4%) patients and peritoneal effusion in 5 (23.8%), which subsided at 1-month follow-up. No complication above grade 2 was recorded.

We calculated the recurrence rate of patients with different treatment modalities (Table 5). Therapies used for intrahepatic lesions included hepatic resection in 6 (28.6%) patients, systemic chemotherapy in 8 (38.1%), transarterial chemoembolization (TACE) in 8 (38.1%), RFA/WMA in 6 (28.6%), tyrosine kinase inhibitor (TKI) therapy in 5 (23.8%), and immunotherapy using programmed death 1 receptor in 1 (4.8%). For the subsequent treatment, 8 (38.1%), 5 (23.8%), 5 (23.8%), 1 (4.8%), and 1 (4.8%) patient received systemic chemotherapy, TKI therapy, immunotherapy, TACE, and RFA, respectively. In postoperative treatments, local therapies (TACE, and RFA) were used after recurrence has been identified. We additionally calculated whether the distance from the lesion to the blood vessel was associated with recurrence. Since the sample size of each treatment was small, this result was only a preliminary finding, and recurrence-related factors requires more sample size data. Mean lesion distances from hepatocaval confluence of patients with recurrence vs. patients without recurrence were 0.61 ± 0.50 cm vs. 0.81 ± 0.64 cm (*p* = 0.47). Mean lesion distances from major hepatic vein of patients with recurrence vs. patients without recurrence were 0.28 ± 0.16 cm vs. 0.16 ± 0.11 cm (*p* = 0.056).

**Figure 1:**
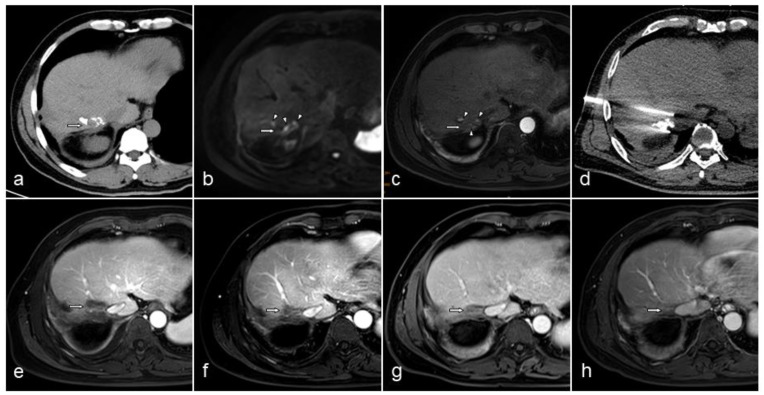
A 55-year-old male patient with hepatocellular carcinoma showing vessel patency after irreversible electroporation (IRE). Eight years ago, the chronic hepatitis B-infected liver mass was detected, surgically resected, and pathologically diagnosed to be hepatocellular carcinoma. Radiofrequency ablation and transarterial chemoembolization (TACE) was performed 4 years and 1 month ago, respectively, for tumor recurrence. IRE was then performed as the lesion was found active. (**a**) Computed tomography (CT) showed iodide deposition in the lesion 1 month after TACE. (**b**) Diffusion-weighted imaging showed a high signal in the lesion after TACE (arrow). (**c**) Contrast-enhanced T1-weighted image (T1WI) showed partial enhancement in the arterial phase (arrowhead). (**d**) CT image during the IRE procedure showed the probes in place and ablation zone. (**e**–**h**) Contrast-enhanced T1WI image in the venous phase showed hepatic vein and postcava remained patent, and the lesion gradually shrank to almost invisible at 1, 3, 6, and 12 months post procedure.

Contrast-enhanced CT or MRI was performed during follow-up to evaluate lesion status (Figure 1). The inferior vena cava remained unobstructed in all patients (Table 6). In one patient, the lesion temporarily blocked the adjacent hepatic vein after ablation, and its patency restored 1 month later. A patient with cholangiocarcinoma developed biliary obstruction and received percutaneous transhepatic cholangiodrainage followed by IRE; however, the symptoms of biliary obstruction were still present after IRE. The changes in the lesions were analyzed on CT or MRI images. The maximum diameter of the lesion shrank to 68% (50–84%), 49% (27–61%), and 38% (25–59%) at 1, 3, and 6 months after ablation, respectively. PFS of the patients with recurrence was 121 (25–566) days after IRE procedure, and the median OS was 451.5 (25–716) days. Four (19.0%) patients died during follow-up. Local recurrence (maximum diameter, 3.04 cm) was observed in 1 (4.8%) patient on Day 176, and distant recurrence was observed in 8 (38.1%) patients (4 lesions located along the electrode path). The median time to distant recurrence was 127 (32–566) days.

## 4. Discussion

Surgical resection is the preferred treatment for primary or secondary intrahepatic malignancies, and complete surgical resection is associated with better prognosis. However, due to the advanced stage at diagnosis or severe trauma post operation, most patients are ineligible for surgical resection, leaving a surgical resection rate as low as 10–25% [15]. In addition, surgical resection can be difficult and cause serious complications for tumors in challenging areas. These tumors include those invading the diaphragm, extensively adhered to the adjacent gastrointestinal tract, and located in the caudate lobe, hepatic hilar area, hepatocaval confluence, etc. Therefore, when surgery is not appropriate, local treatment can serve as effective alternatives, such as TACE, RFA, MWA, percutaneous ethanol injection, and radiotherapy. Heat-based ablation sometimes faces limitations when the tumors are near large blood vessels, bile duct, stomach, or the intestines. Although artificial pneumoperitoneum or ascites can be created to push the stomach or intestines away, the heat-sink effect of large vessels can seriously affect the treatment outcome [16].

Due to non-thermal mechanism, IRE only destroys tumor cell membranes without damaging the extracellular matrix components. As a result, the structural continuity of blood vessels and bile ducts can be maintained [17]. The efficacy of IRE for HCC was confirmed by complete pathological necrosis after treatment [18]. As the main structural components of the bile ducts, blood vessels, and nerves are fibrous tissue that are short of phospholipid bilayer, IRE rarely causes serious damages to these structures. In addition, IRE is not affected by the heat sink effect, making it especially suitable for ablating malignant tumors adjacent to structures such as blood vessels and bile ducts. 

In this study, the success rate of IRE procedure was 100% without arrhythmia identified using the cardiac synchronization technique, even though the lesions were adjacent to the diaphragm and heart. In O’Neill et al.’s systematic review on 481 patients receiving IRE, only 1.2% (5/422) of those undergoing cardiac synchronization experienced arrhythmias compared with 22.0% (13/59) of those without synchronization [19]. In the present study, 14.3% (3/21) of the patients developed fever after IRE, which resolved within 3 days after antibiotics were given. Meanwhile, 9.5% (2/21) of the patients presented with postoperative pain, with one being NRS grade 2 and the other one grade 3. Govindarajan et al.’s retrospective study on comparing postprocedural pain generated similar results to the current study, given that the mean pain score for HCC treated with IRE (28 sessions) and RFA (25 sessions) was 1.96 and 2.25, respectively [20]. Pleural effusion occurred in 52.4% (11/21) of the studied patients, whereas previous studies revealed that to be 9.5–16% [21,22]. The reason for a higher incidence in this current study is thought to be the closeness of the lesions to the diaphragm and pleura. The immediate postoperative imaging studies showed peritoneal effusion in 23.8% (5/21) of the patients, which subsided spontaneously at 1-month follow-up. Both pleural effusion and peritoneal effusion were self-limiting and did not require further treatment other than close monitoring and follow-up.

In this study, the systemic treatments that some patients received after surgery included systemic chemotherapy (8/21; 38.1%), TKI therapy (5/21; 23.8%), and immunotherapy (5/21; 23.8%). Locoregional therapy included RFA (2/21; 9.5%) and TACE (1/21; 4.8%) for distant recurrence. PFS of the patients with recurrence was 121 (25–566) days after IRE. Four (19.0%) patients died by the end of follow-up, and the median survival time was 451.5 (25–716) days. Some previous studies on liver tumor IRE reported better results. In Mafeld et al.’s bi-institutional analysis on percutaneous IRE for hepatic malignancy [23], 52 patients underwent percutaneous IRE for 59 liver tumors in 53 sessions and had a median survival time of 38 months; moreover, 44% of them were progression-free at 12 months. Although IRE combined with chemotherapy showed a good prognosis in pancreatic cancer [24], studies on liver tumors are not yet sufficient. Of the 40 patients with 77 lesions in Langan et al.’s study, 29% received systemic therapy following IRE. However, only ablation zone size and body mass index were significantly associated with local ablation zone recurrence, and subsequent systemic therapy did not significantly improve the treatment outcomes [25]. Therefore, more clinical studies are required to verify the efficacy of IRE combined with chemotherapy. Since IRE-induced apoptosis can better retain cell antigens than thermal ablation, it is believed that IRE can overcome tumor-associated immunosuppression and promote tumor antigen-specific tissue-resident memory CD8+ T cells to more efficiently activate the immune response. Under the circumstances, the combination therapy of IRE and immunotherapy for tumor has become a hot topic in recent years [26,27,28]. Alnaggar et al. included 40 patients with stage IV HCC and equally divided them into two groups: the IRE group and the IRE plus allogenic natural killer (NK) cell immunotherapy group. The IRE + NK group generated a longer median OS than the IRE group did (10.1 vs. 8.9 months) [29]. In addition, preclinical studies have confirmed the enhanced gene transfer of IRE that leads to both local and systemic immune response [30,31]. Overall, IRE and systemic therapy, especially immunotherapy, showed synergetic effect when combined. Despite its great potential in treating cancer, more clinical evidence is needed to further support this perspective. 

The changes in the lesions were monitored by contrast-enhanced CT or MRI. The maximum diameter of the lesion shrank to 68% (50–84%), 49% (27–61%), and 38% (25–59%) at 1, 3, and 6 months after ablation. The ablated lesions decreased in size rapidly within 3 months after IRE and shrank slowly over the following 3 months. A case report by Kasivisvanathan V et al. demonstrated a solitary chemoresistant liver metastasis at the porta hepatis that decreased in tumor volume from 5.25 cm^3^ to 3.16 cm^3^ after IRE without early or late complications [32]. Similar results were illustrated in a study on perivascular hepatic malignant tumors treated with IRE, where the median ablation zone area decreased to 9 cm^2^, 2.3 cm^2^, and 2.3 cm^2^ at 1, 3, and 6 months [33]. When compared to tumors treated with MWA, liver tumors treated with IRE underwent faster involution, but liver enzymes levels were comparable [34]. These findings implied that IRE usually requires a short recovery period. 

The present study suggested IRE as an effective technique for unresectable primary or secondary liver tumors at the hepatocaval confluence while thermal ablation produces suboptimal outcomes due to the heat sink effect. A retrospective study reviewing 28 patients and 65 perivascular tumors treated with IRE had an overall morbidity of 3% with 1 (1.9%) tumor being persistent disease and 3 (5.7%) tumors recurred locally [33]. In another study reporting 43 malignant liver tumors located near the major portal or hepatic veins being treated with IRE, intrahepatic tumor recurrence was observed in 13 (33%) of the 40 completely ablated tumors at 2–18 months, and only 2 (15%) of the 13 were found in the ablation zone [15]. However, although studies investigating local recurrence after RFA reported an average recurrence rate of 10–30% [35], the number could increase to 48–58% when the tumors were located at major blood vessels or in challenging areas [36,37,38]. Therefore, IRE has prominent advantages over RFA in lowering local recurrence rate for perivascular tumors. Speaking of the factors affecting local recurrence of IRE, a tumor diameter > 4 cm is a possible reason that increases local recurrence rate [39]. Given the fact that needle tract ablation is not possible with current IRE equipment, needle tract seeding is a predictable complication of a non-thermal treatment method. In a cohort study, needle tract seeding was observed in 27.5% (11/40) of the completely ablated lesions [15]. The resulting overall local recurrence rate (42.6%) is comparable to the published recurrence rates of 6–55% (median, 29%) [33,40,41,42]. When the lesions were close to the hepatocaval confluence, the electrode path was longer than that of the lesions located more superficially, which could potentially increase the risk of needle tract seeding. This might explain the higher distant recurrence rate in the current study.

## 5. Conclusions

Although more data is needed to solidify the indications for using IRE in treating hepatic tumors at the hepatocaval confluence, its safety profile and patient sustainable rate has endorsed IRE to be a safe and feasible treatment option when thermal ablation is not appropriate. In addition, high success and low local recurrence rates foreshadow the efficacy of IRE, and these results are to be validated over a longer follow-up time in a greater number of patients. Synergistic therapy may be the trend of IRE to further benefit patients.

## Figures and Tables

**Table 1:** Patient demographics.

Variable	Data
Total patient, *n*	21
Median follow-up, d, median (range)	313 (25–1786)
Tumors treated per patient, *n*, median (range)	1 (1–5)
Age, y, median (range)	58 (41–83)
Sex, *n* (%)	
Male	16 (76.2%)
Female	5 (23.8%)
BMI (kg/m^2^), median (range)	22.9 (15.6–26.0)
Diabetes mellitus, *n* (%)	3 (14.3%)
Hypertension, *n* (%)	11 (52.4%)
Heart disease, *n* (%)	4 (19.0%)
Cirrhosis, *n* (%)	9 (42.9%)
Child-Pugh class	
A	20 (95.2%)
B	1 (4.8%)
C	0 (0.0%)

Note: BMI, body mass index; TKI, tyrosine kinase inhibitor.

**Table 2:** Characteristics of lesions at the hepatocaval confluence.

Variable	Data
Tumor size	
Median maximum diameter, cm (range)	1.81 (1.31–4.04)
1.01–2.00 cm, *n* (%)	12 (57.1%)
2.01–3.00 cm, *n* (%)	5 (23.8%)
≥3.01 cm, *n* (%)	4 (19.1%)
Location of tumor, *n* (%)	
Segments 2	1 (4.8%)
Segments 4a	2 (9.5%)
Segments 7	7 (33.3%)
Segments 8	8 (38.1%)
Junctional region of segments	3 (14.3%, 2 × (S4a + S8), 1 × (S7 + S8))
Distance from the hepatocaval confluence, cm	
Median distance (range)	0.50 (0.10–1.72)
≤0.50, *n* (%)	11 (52.4%)
0.50–1.00, *n* (%)	4 (19.0%)
1.01–2.00, *n* (%)	6 (28.6%)
Distance from the major hepatic vein, cm	
Median distance (range)	0.15 (0.10–0.45)
Lesions distant from the hepatocaval confluence	
Yes, *n* (%), median (range)	8 (38.1%), 1.5 (1–4)
None, *n* (%)	13 (61.9%)
Primary tumor types	
Hepatocellular carcinoma, *n* (%)	9 (42.9%)
Cholangiocarcinoma, *n* (%)	2 (9.5%)
Intestinal cancer, *n* (%)	4 (19.1%)
Gastric carcinoma, *n* (%)	3 (14.3%)
Pancreatic carcinoma, *n* (%)	3 (14.3%)

**Table 3:** Technical details of IRE procedure.

Variable	Data
Treated tumors, *n*	38
Lesions at the hepatocaval confluence, *n*, patients, *n* (%)	21, 21 (100%)
Lesions distant from the hepatocaval confluence, *n*, patients, *n* (%)	17, 8 (38.1%)
Procedures, *n*	21
Number of electrodes, median (range)	4 (2–4)
2, *n* (%)	8 (38.1%)
3, *n* (%)	2 (9.5%)
4, *n* (%)	11 (52.4%)
Exposure length of electrodes (cm), median (range)	2.25 (1.5–3)
Spacing (cm), median (range)	1.9 (1.5–2.2)
Electrode replacement	
Pull-back technique, *n* (%)	14 (66.7%)
Electrode replacement, *n* (%)	6 (28.6%)
IRE ablation size	
Median largest diameter, cm (range)	3.87 (2.85–6.55)
2.01–3.00, *n* (%)	2 (9.5%)
3.01–4.00, *n* (%)	10 (47.6%)
4.01–5.00, *n* (%)	5 (23.8%)
≥5.01, *n* (%)	4 (19.0%)
IRE ablation size/tumor size, median (range)	2.83 (1.4–3.2)
Patients with lesions distant from the hepatocaval confluence receiving IRE, *n* (%)	4 (19.0%, 6 lesions)
Patients with lesions distant from the hepatocaval confluence receiving thermal ablation, *n* (%)	4 (19.0%, 11 lesions)

**Table 4:** Acute complications.

Variable	Data
CIRSE grade	
1	
Fever, *n* (%)	3 (14.3%)
Pain, *n* (%)	2 (9.5%, 1 × Grade 2, 1 × Grade 3)
2	
Hydrothorax, *n* (%)	11 (52.4%)
Seroperitoneum, *n* (%)	5 (23.8%)
3	0
4	0
5	0

**Table 5:** Treatment and recurrence.

Variable	Patient Number, *n* (%)	Recurrence, *n* (%)	Recurrence without This Treatment, *n* (%)
Prior treatments			
Hepatic resection, *n* (%)	6 (28.6%)	2 (33.3%)	7 (46.7%)
Systemic chemotherapy, *n* (%)	8 (38.1%)	3 (37.5%)	6 (46.2%)
Hepatic arterial therapy, *n* (%)	8 (38.1%)	4 (50%)	5 (38.5%)
Radiofrequency ablation OR Microwave ablation, *n* (%)	6 (28.6%)	4 (66.7%)	5 (33.3%)
TKI Targeted Therapies, *n* (%)	5 (23.8)	4 (80%)	5 (29.4%)
Immunotherapy, *n* (%)	1 (4.8%)	1 (100%)	8 (40%)
Three-dimensional conformal radiation, *n* (%)	0 (0.0%)	-	-
Postoperative treatments			
Systemic chemotherapy	8 (38.1%)	5 (62.5%)	4 (30.8%)
Hepatic arterial therapy (TACE)	1 (4.8%)	1 (100%)	8 (40%)
TKI Targeted Therapies	5 (23.8%)	4 (80%)	5 (31.2%)
Immunotherapy	5 (23.8%)	1 (20%)	8 (50%)
Radiofrequency ablation	1 (4.8%)	1 (100%)	8 (40%)
Hepatic resection	0 (0.0%)	-	-
Three-dimensional conformal radiation	0 (0.0%)	-	-

**Table 6:** Postoperative results.

Variable	Data
Postcaval status	
Vein patent	21 (100%)
Postcava occluded	0 (0.0%)
Hepatic vein status	
Vein patent	20 (95.2%)
Vein occluded	1 (4.8%)
Bile duct status	
Bile duct patent	20 (95.2%)
Bile duct occluded	1 (4.8%)
Ablation size at 1 mo to postoperative size, median (range)	0.68 (0.50–0.84)
Ablation size at 3 mo to postoperative size, median (range)	0.49 (0.27–0.61)
Ablation size at 6 mo to postoperative size, median (range)	0.38 (0.25–0.59)
Progression-free survival (d), median (range)	121 (25–566)
Death, *n* (%), survival time (d), median (range)	4 (19.0%), 451.5 (25–716)
Disease persistence/recurrence	
Local recurrence, *n* (%), time to recurrence (d)	1 (4.8%), 176
Distant recurrence, *n* (%), time to recurrence (d), median (range)	8 (38.1%), 127 (32–566)

## Data Availability

The dataset used and/or analyzed during the current study are available from the corresponding author on reasonable request.
